# Supplementary material for: Functional characterization of three flavonoid glycosyltransferases from Andrographis paniculata
Source: R Soc Open Sci. 2019 Jun 12;6(6):190150. doi: 10.1098/rsos.190150 (PMC6599797; doi:10.1098/rsos.190150)
Supplement: UPLC-Q-TOF-MS characterization data [file rsos190150supp1.docx]

Supplementary data file

**Functional characterization of three flavonoid glycosyltransferases from *Andrographis paniculata***

Yuan Li^1,2^, Xin-Lin Li^4^, Chang-Jiang-Sheng Lai^2^, Rui-Shan Wang^2^, Li-Ping Kang^2^, Ting Ma^1^, Zhen-Hua Zhao^1^, Wei Gao^*,3,4,5^ and Lu-Qi Huang^*,1,2^

1. Shandong University of Traditional Chinese Medicine, Jinan, 250355, P. R. China.

2. National Resource Center for Chinese Materia Medica, China Academy of Chinese Medical Sciences, Beijing 100700, P. R. China.

3. School of Pharmaceutical Sciences, Capital Medical University, Beijing 100069, P. R. China.

4. School of Traditional Chinese Medicine, Capital Medical University, Beijing 100069, P. R. China.

5. Advanced Innovation Center for Human Brain Protection, Capital Medical University, Beijing 100069, P. R. China.

*Corresponding author: Wei Gao; e-mail: weigao@ccmu.edu.cn; Tel.: +86 10 83916572; Fax:+86 10 83911627.

Lu-Qi Huang; e-mail: [huangluqi01@126.com](mailto:huangluqi01@126.com); Tel: +86 10 8404 4340, Fax: +86 10 8402 7175

##
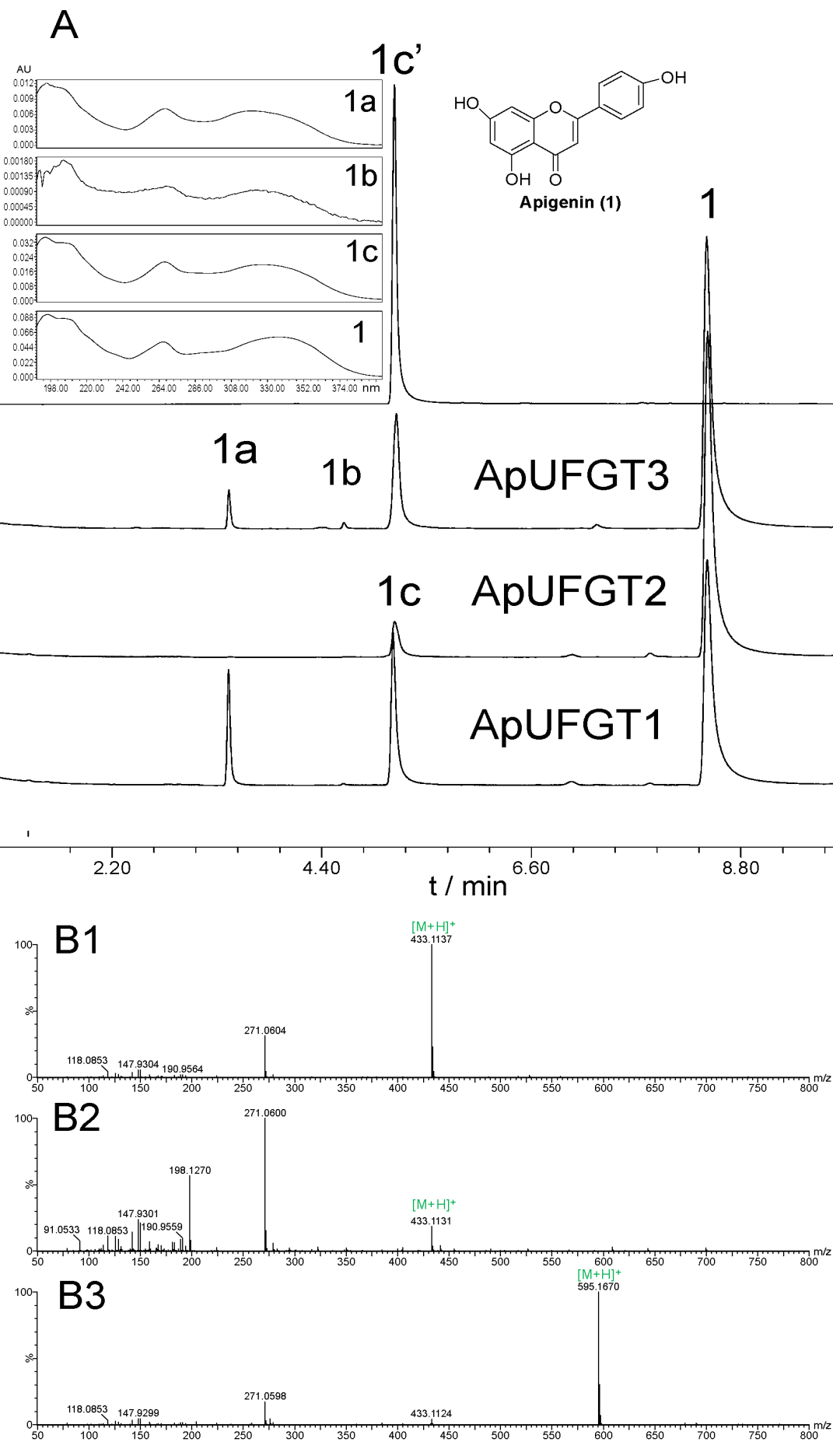


## Fig. S1 UPLC-Q-TOF-MS analysis of ApUFGTs enzyme products using Apigenin (1) as an aglycon acceptor. (A) UPLC chromatogram and UV spectra of 1 and enzyme products 1a ,1b and 1c. Peak for the authentic standard apigetrin is 1c’; (B1), (B2), and (B3) Typical positive ion MS spectra for 1c,1b and 1a.

##
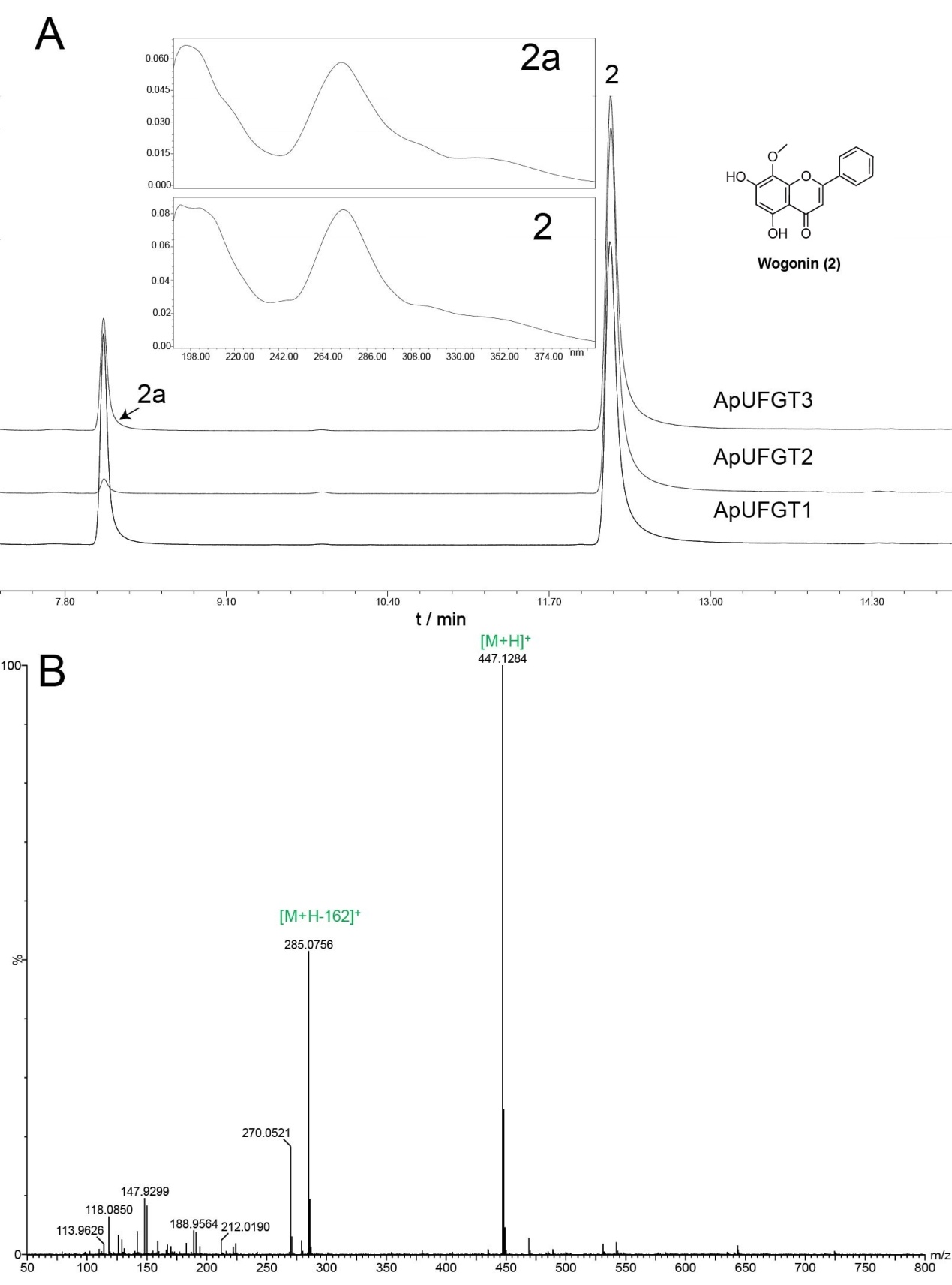


## Fig. S2 UPLC-Q-TOF-MS analysis of ApUFGTs enzyme product using Wogonin (2) as an aglycon acceptor. (A) UPLC chromatogram and UV spectra of 2 and enzyme product 2a; (B) Typical positive ion MS spectra for 2a.

##
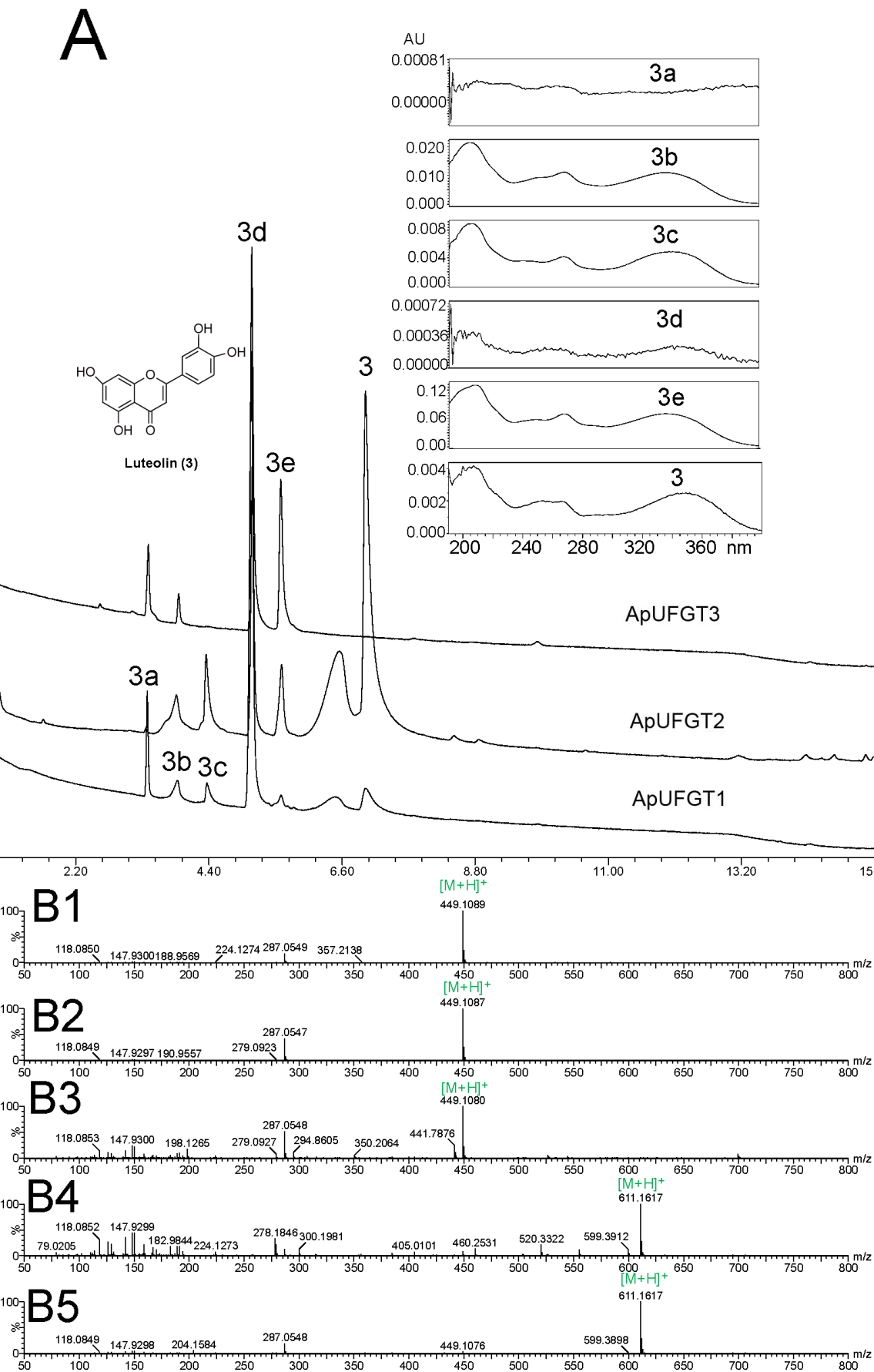


## Fig. S3 UPLC-Q-TOF-MS analysis of ApUFGTs enzyme products using Luteolin (3) as an aglycon acceptor. (A) UPLC chromatogram and UV spectra of 3 and enzyme products 3a, 3b, 3c, 3d and 3e; (B1), (B2), (B3), (B4), and (B5) Typical positive ion MS spectra for 3e, 3d, 3c, 3b and 3a.

##
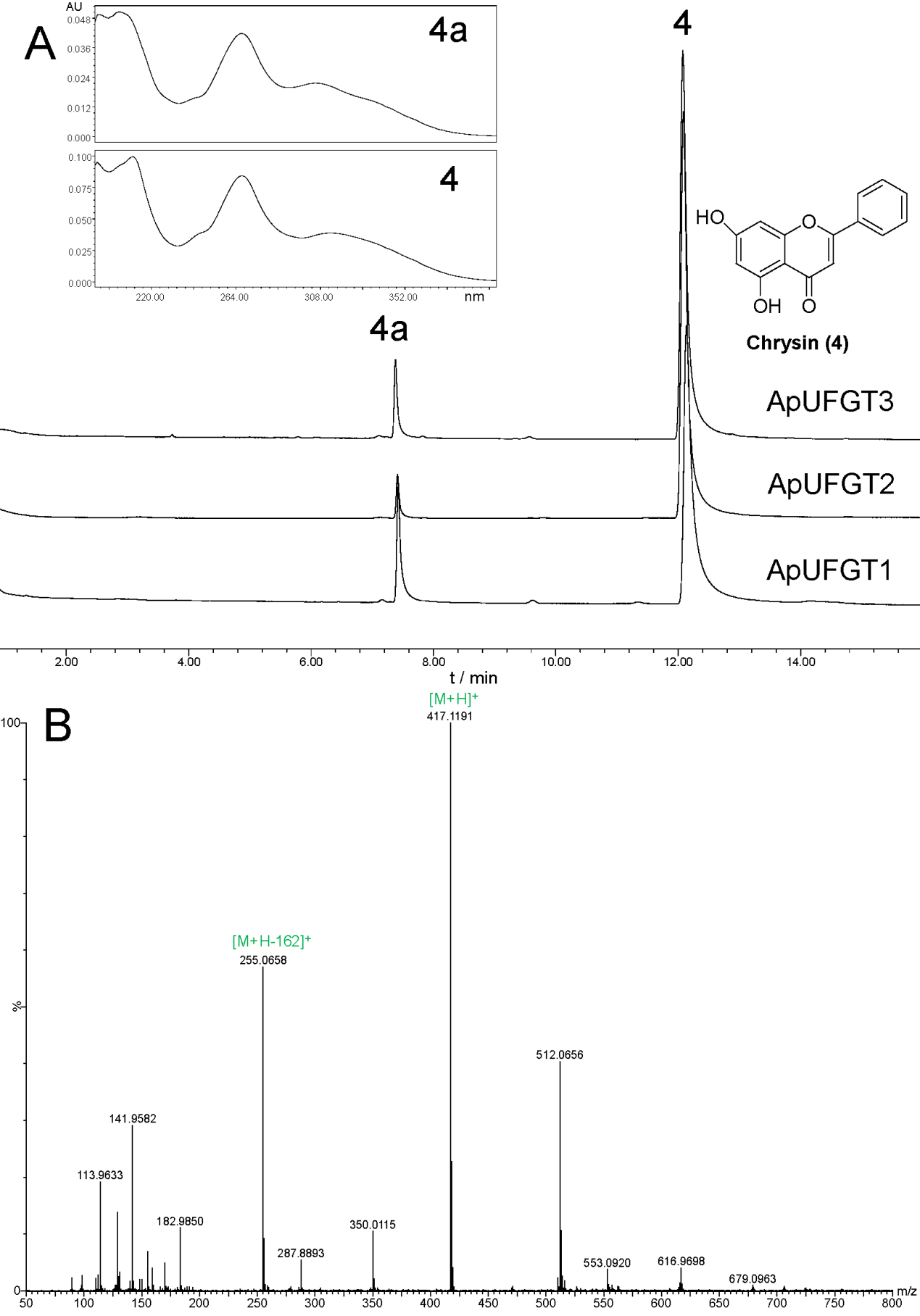


## Fig. S4 UPLC-Q-TOF-MS analysis of ApUFGTs enzyme product using Chrysin (4) as an aglycon acceptor. (A) UPLC chromatogram and UV spectra of 4 and enzyme product 4a; (B) Typical positive ion MS spectra for 4a.

##
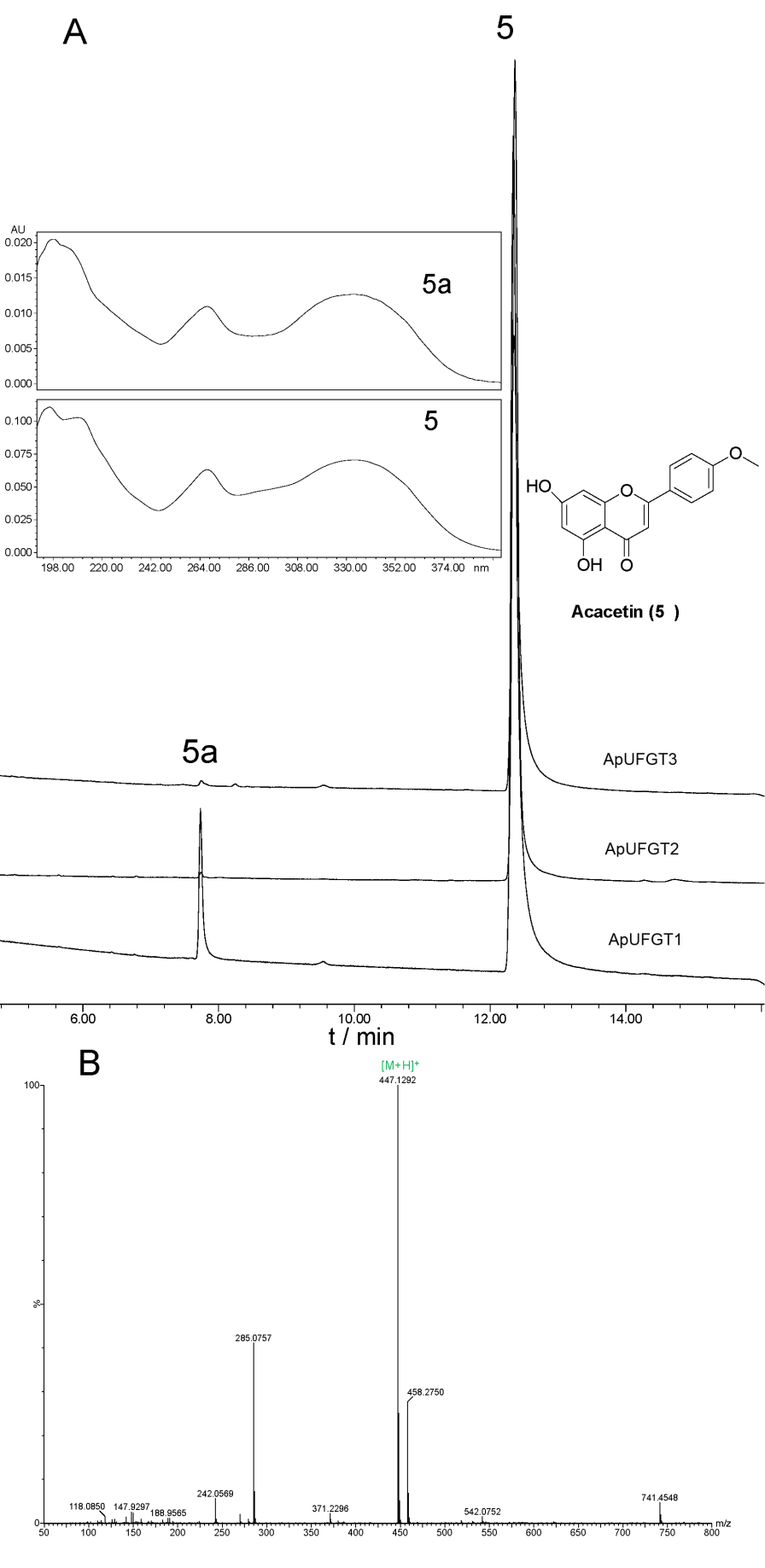


## Fig. S5 UPLC-Q-TOF-MS analysis of ApUFGTs enzyme product using Acacetin (5) as an aglycon acceptor. (A) UPLC chromatogram and UV spectra of 5 and enzyme product 5a; (B) Typical positive ion MS spectra for 5a.

##
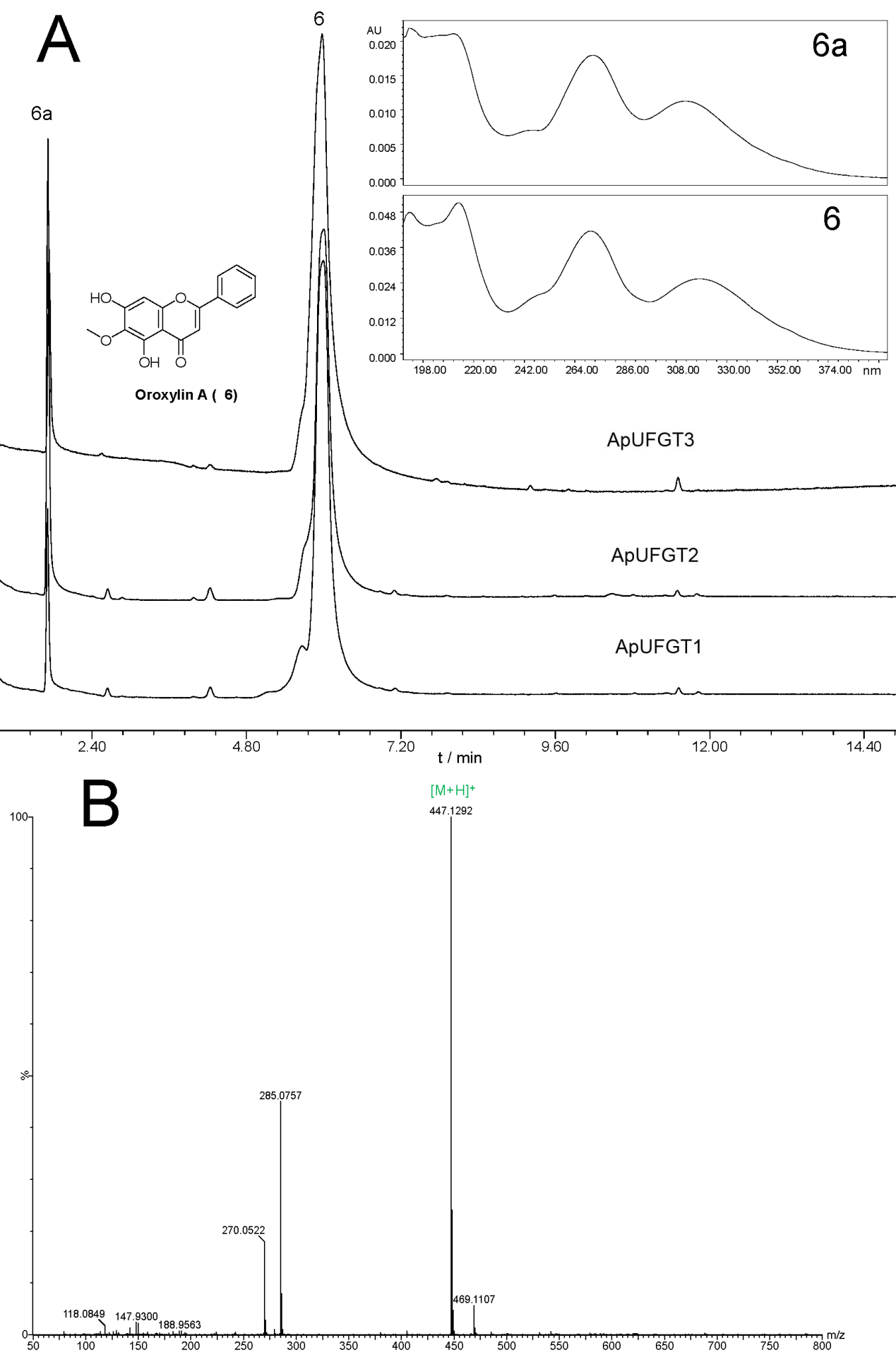


## Fig. S6 UPLC-Q-TOF-MS analysis of ApUFGTs enzyme product using Oroxylin A (6) as an aglycon acceptor. (A) UPLC chromatogram and UV spectra of 6 and enzyme product 6a; (B) Typical positive ion MS spectra for 6a.

##
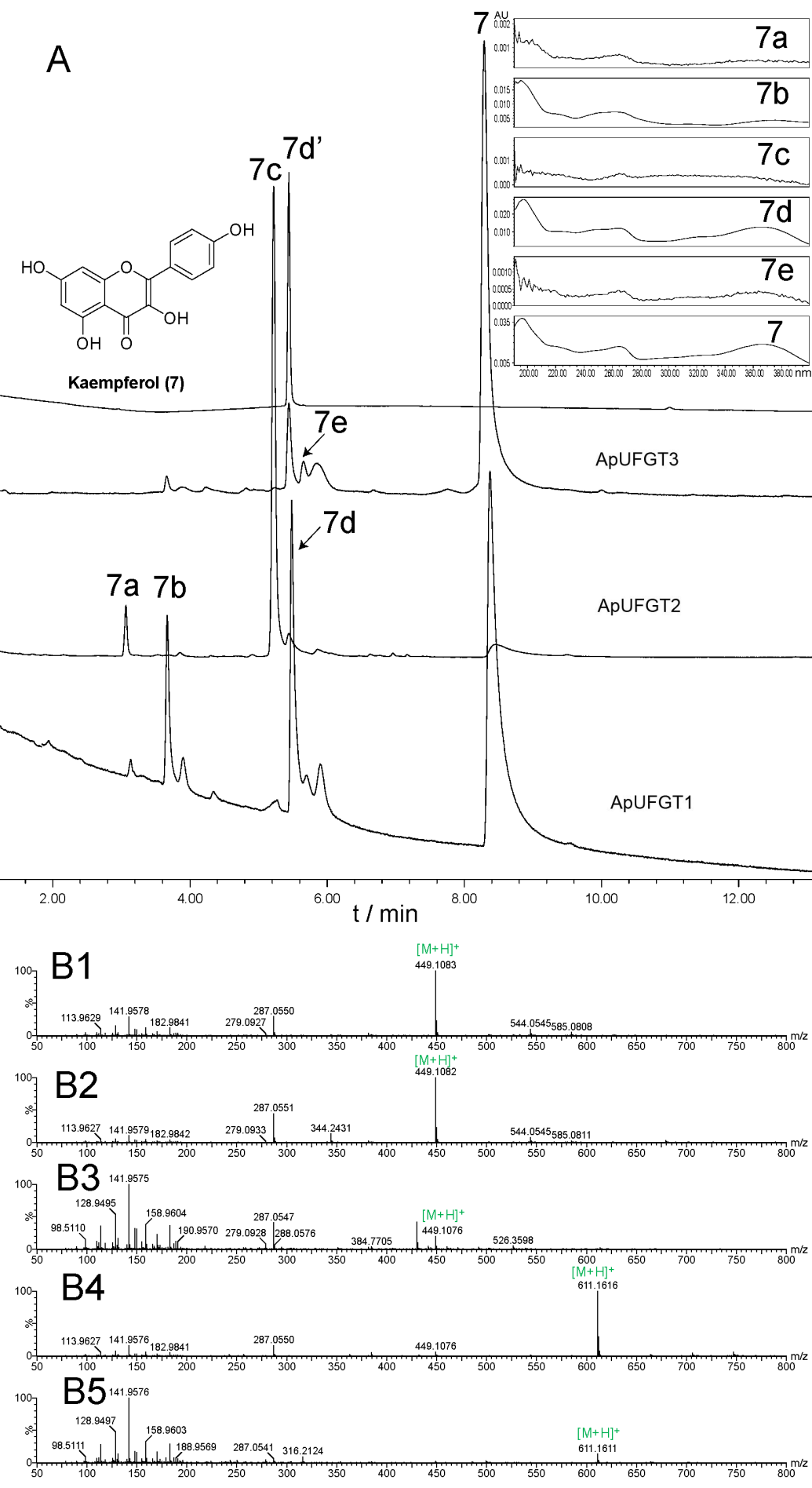


## Fig. S7 UPLC-Q-TOF-MS analysis of ApUFGTs enzyme products using Kaempferol (7) as an aglycon acceptor. (A) UPLC chromatogram and UV spectra of 7 and enzyme products 7a, 7b, 7c, 7d and 7e. Peak for the authentic standard populnin is 7d’; (B1), (B2), (B3), (B4), and (B5) Typical positive ion MS spectra for 7e, 7d, 7c, 7b and 7a.

##
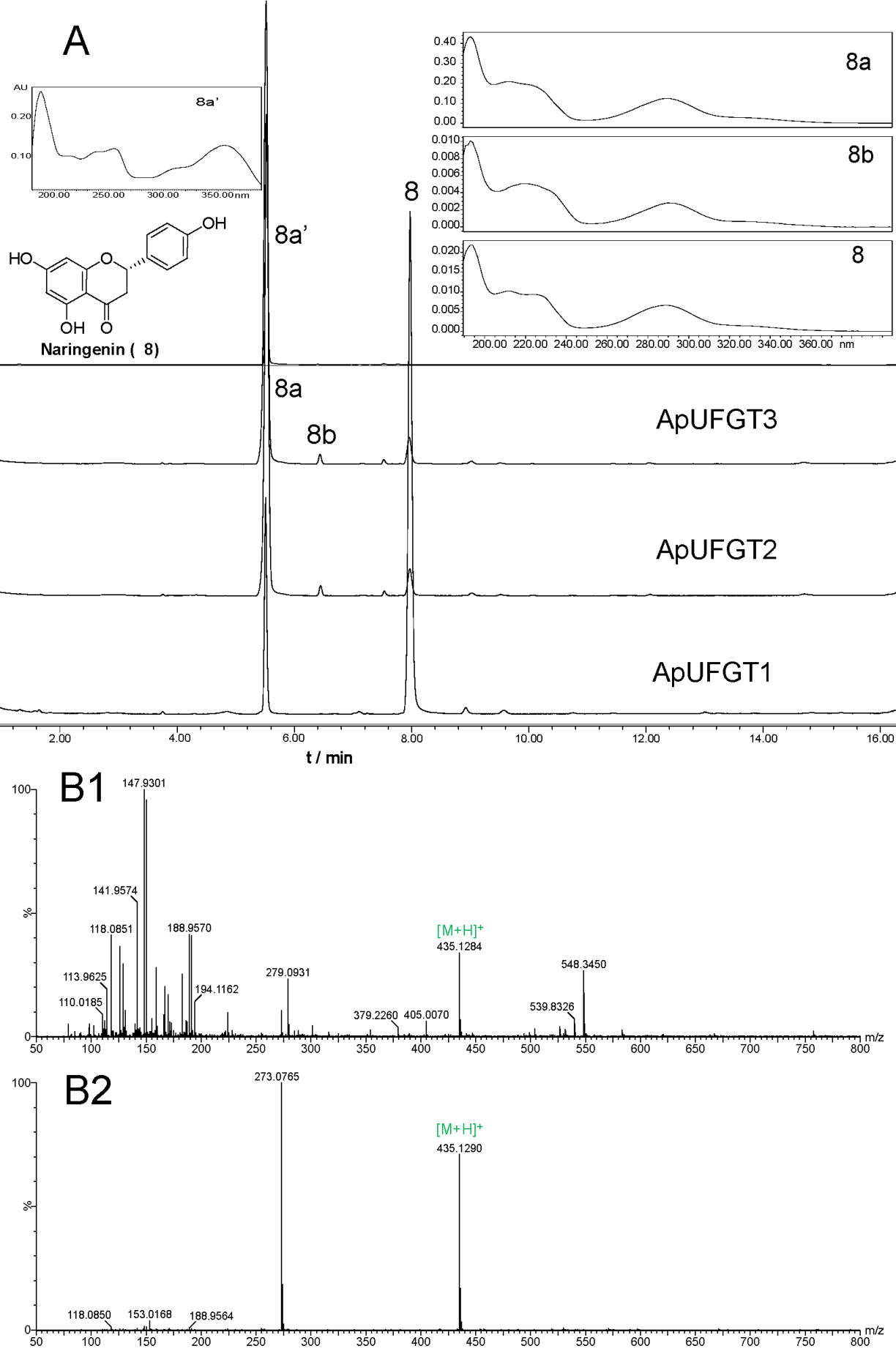


## Fig. S8 UPLC-Q-TOF-MS analysis of ApUFGTs enzyme products using Naringenin (8) as an aglycon acceptor. (A) UPLC chromatogram and UV spectra of 8 and enzyme products 8a and 8b. Peak for the authentic standard prunin is 8a’; (B1) and (B2) Typical positive ion MS spectra for 8b and 8a.

##
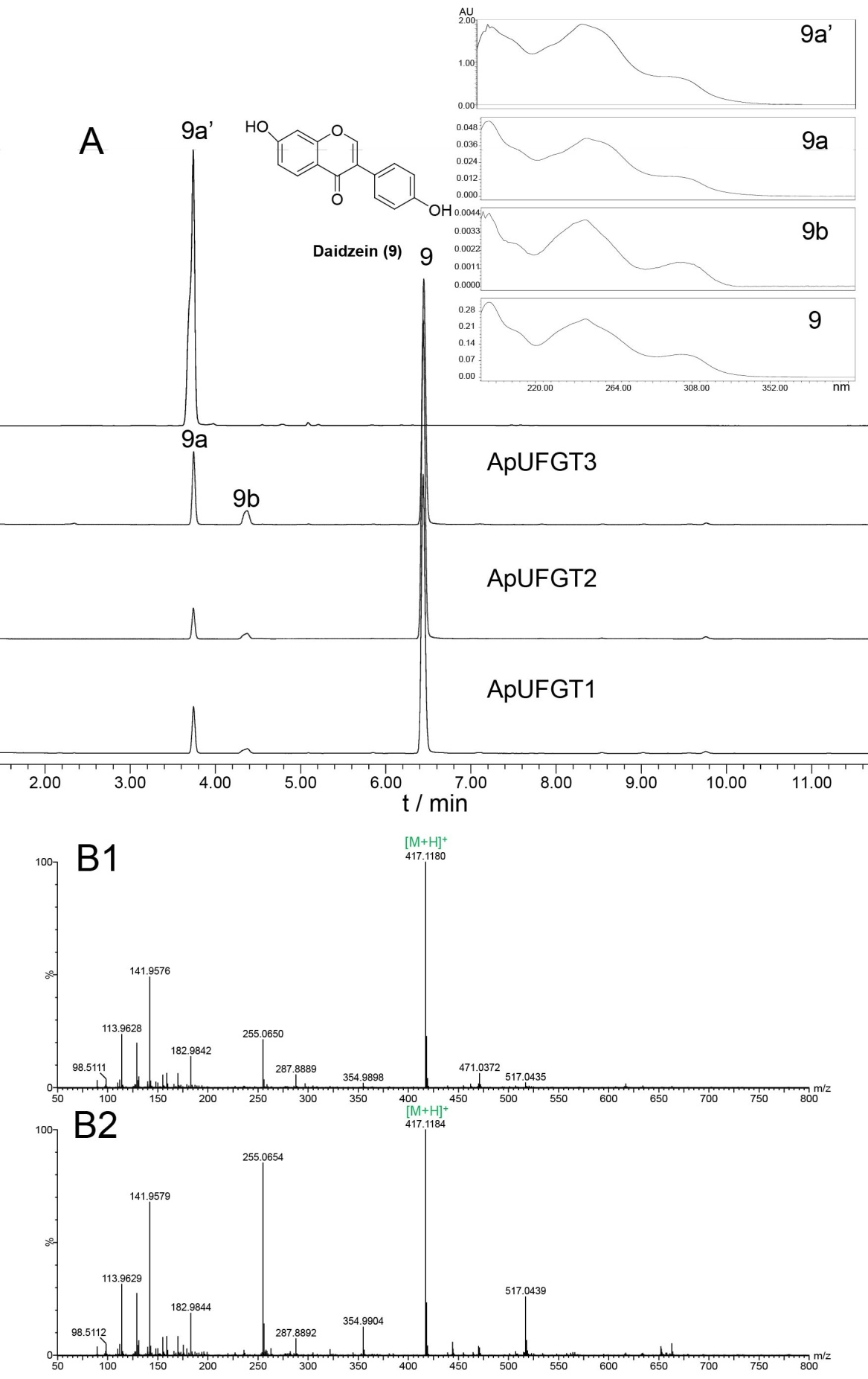


## Fig. S9 UPLC-Q-TOF-MS analysis of ApUFGTs enzyme products using daidzein (9) as an aglycon acceptor. (A) UPLC chromatogram and UV spectra of 9 and enzyme products 9a and 9b. Peak for the authentic standard daidzin is 9a’; (B1) and (B2) Typical positive ion MS spectra for 9b and 9a.

##
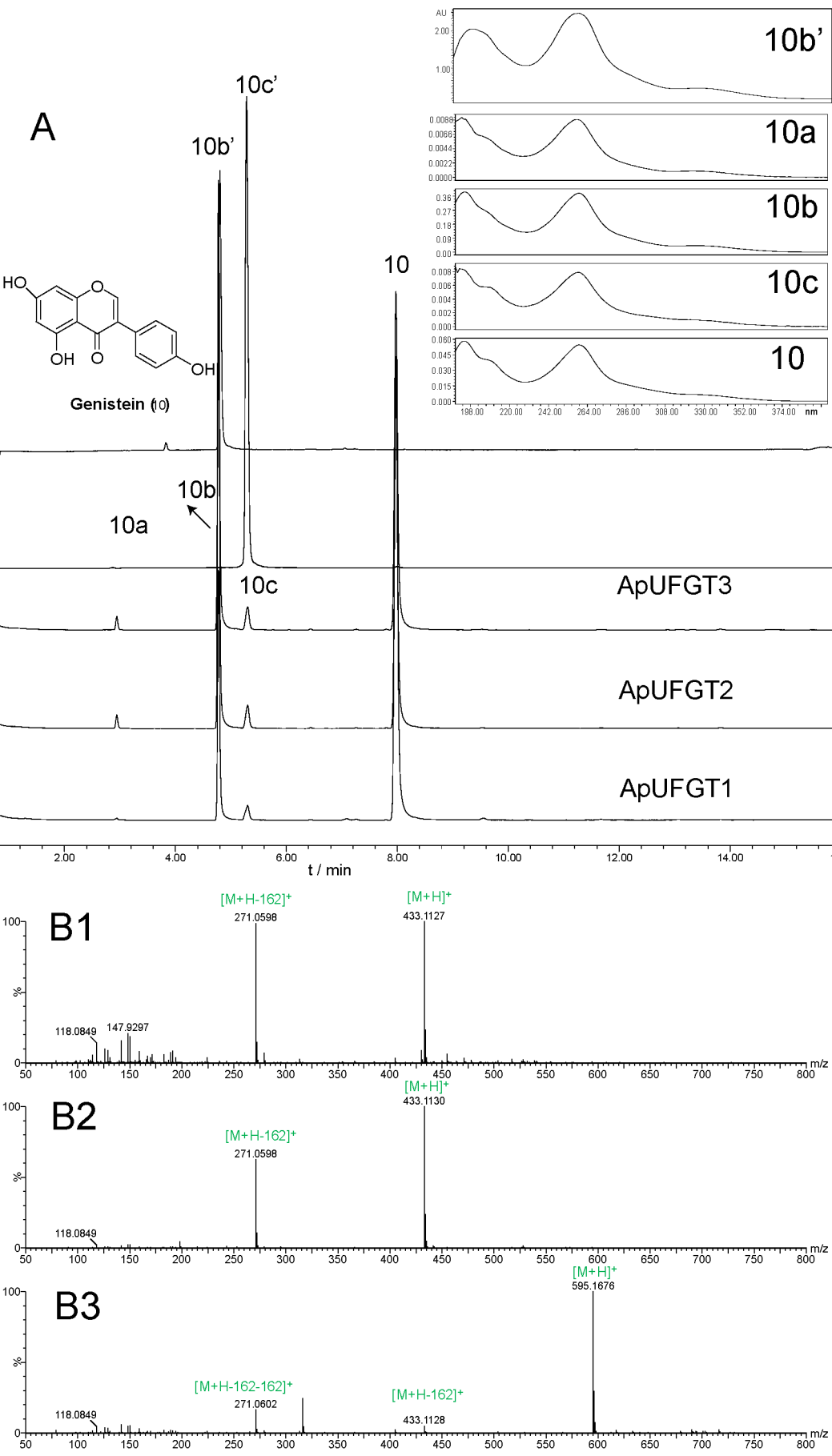


**Fig. S10** UPLC-Q-TOF-MS analysis of ApUGT enzyme products using genistein (**10**) as an aglycon acceptor. (A) UPLC chromatogram and UV spectra of **10** and enzyme products **10a, 10b, 10c**. Peak for the authentic standard genistin is **10b’**, peak for the authentic standard sophoricoside is **10c’**; (B1), (B2), and (B3) Typical positive ion MS spectra for **10c, 10b** and **10a**.

##
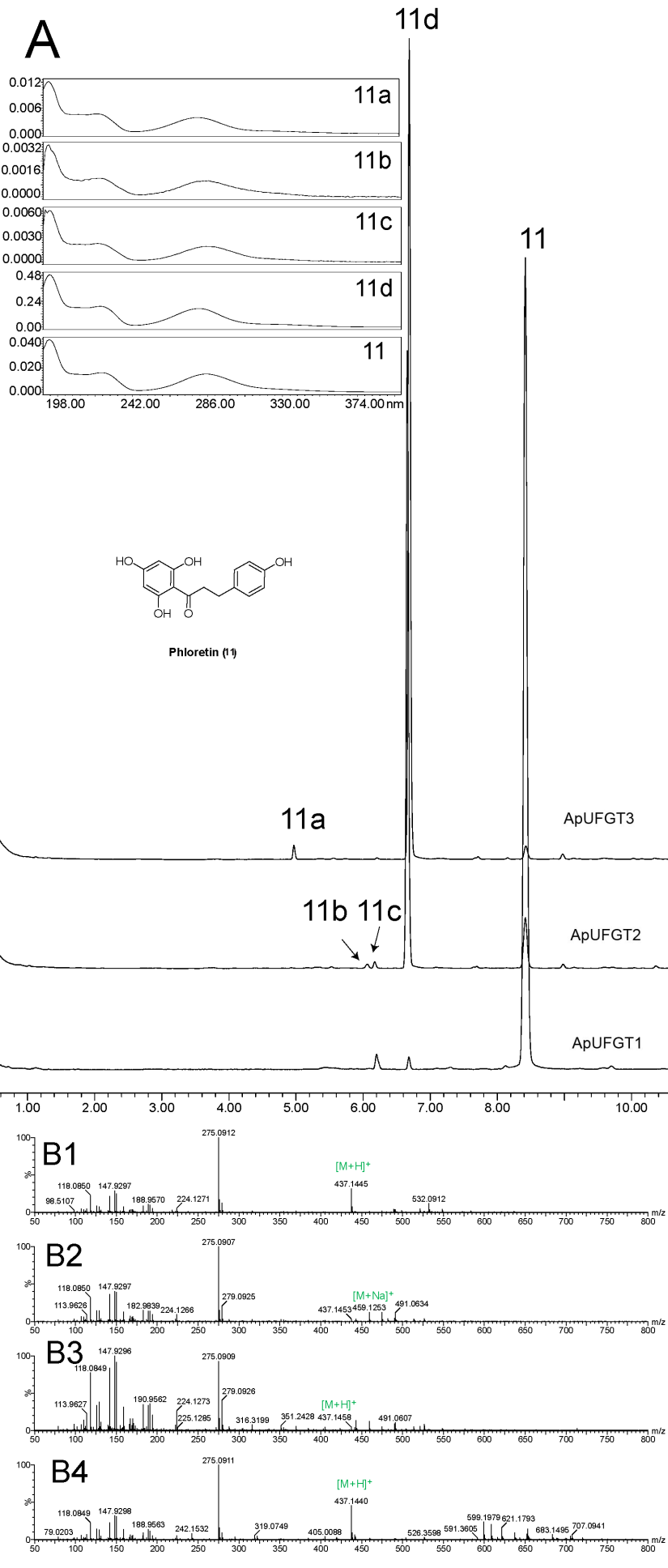


## Fig. S11. UPLC-Q-TOF-MS analysis of ApUFGTs enzyme products using phloretin (11) as an aglycon acceptor. (A) UPLC chromatogram and UV spectra of 11 and enzyme products 11a, 11b, 11c and 11d; (B1), (B2), (B3), and (B4) Typical positive ion MS spectra for 11d, 11c, 11b and 11a.

##
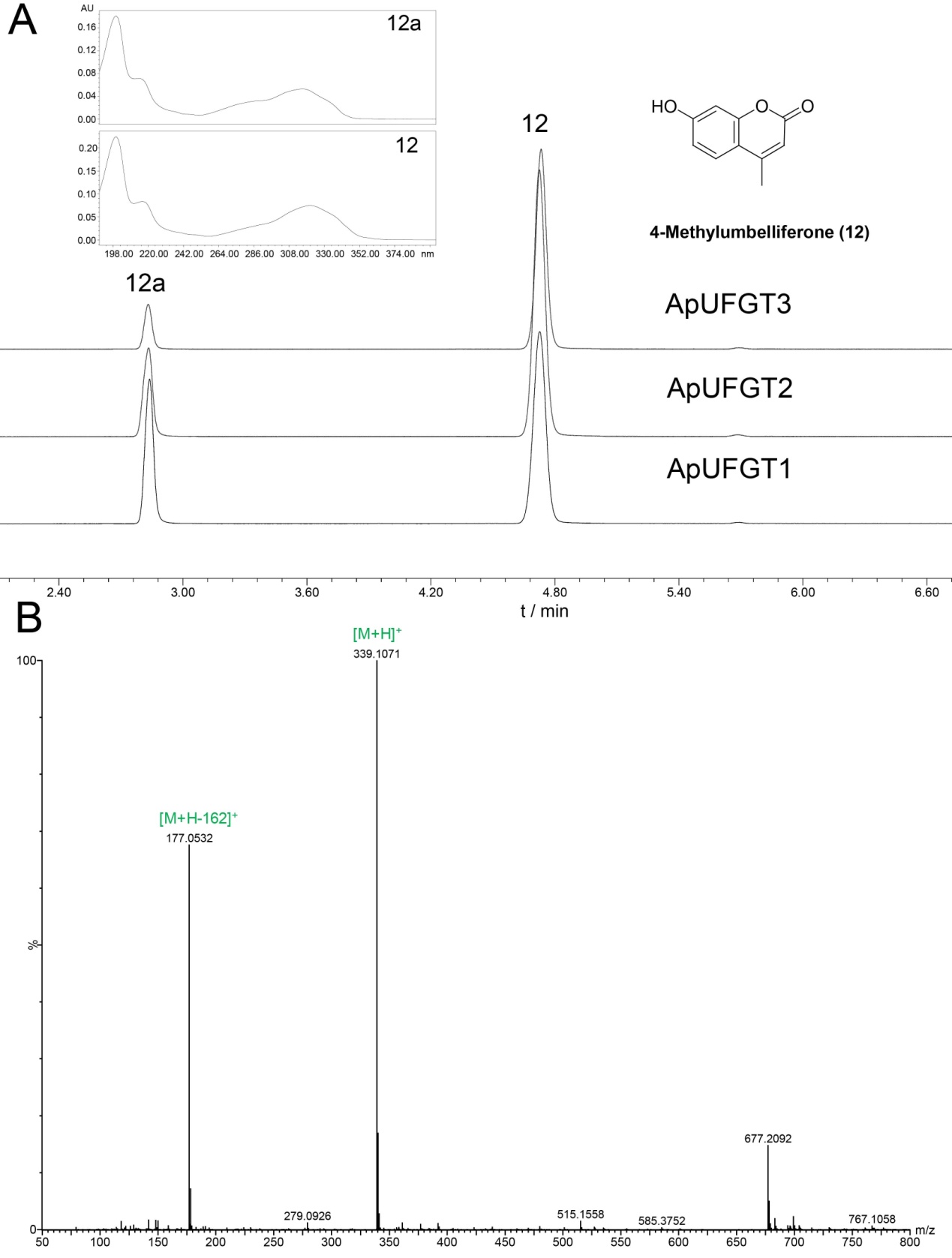


## Fig. S12 UPLC-Q-TOF-MS analysis of ApUFGTs enzyme product using 4-Methylumbelliferone (12) as an aglycon acceptor. (A) UPLC chromatogram and UV spectra of 12 and enzyme product 12a; (B) Typical positive ion MS spectra for 12a.

##
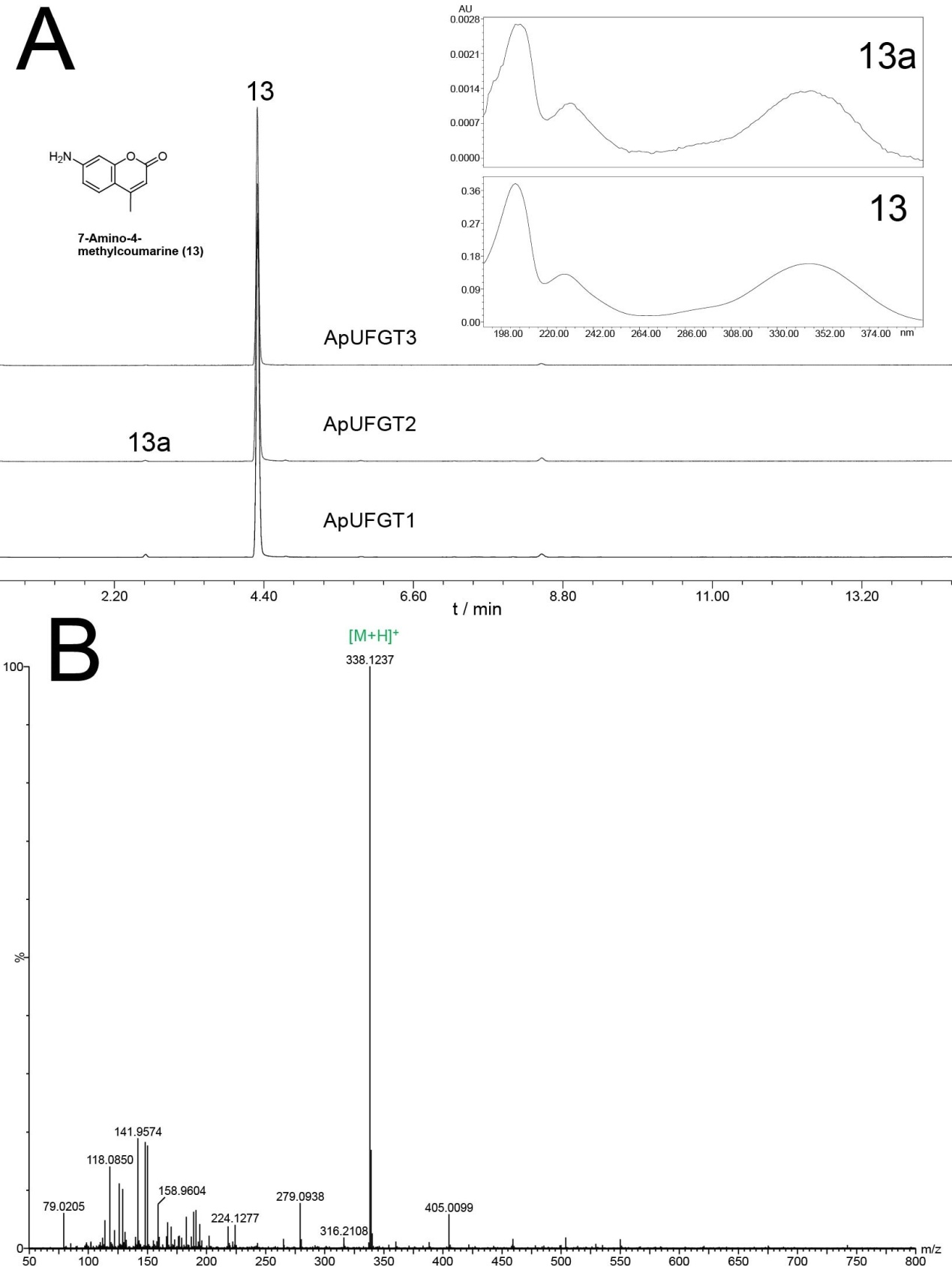


## Fig. S13 UPLC-Q-TOF-MS analysis of ApUFGTs enzyme product using 7-Amino-4-

## methylcoumarine (13) as an aglycon acceptor. (A) UPLC chromatogram and UV spectra of 13 and enzyme product 13a; (B) Typical positive ion MS spectra for 13a.

##
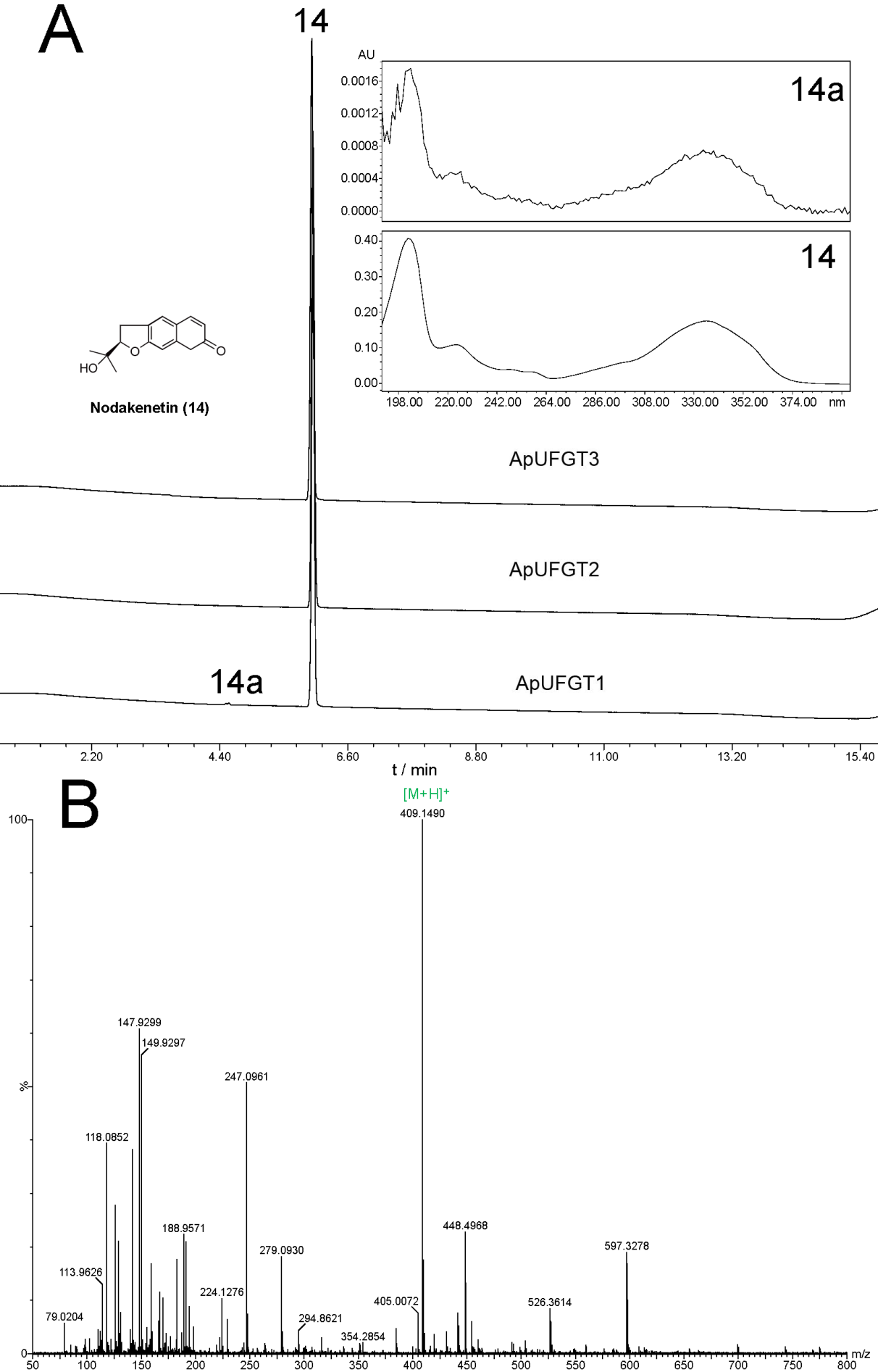


## Fig. S14 UPLC-Q-TOF-MS analysis of ApUFGTs enzyme product using Nodakenetin (14) as an aglycon acceptor. (A) UPLC chromatogram and UV spectra of 14 and enzyme product 14a; (B) Typical positive ion MS spectra for 14a.
